# Blood Culture-Negative Infective Endocarditis by *Mycoplasma hominis*: Case Report and Literature Review

**DOI:** 10.3390/jcm11133841

**Published:** 2022-07-02

**Authors:** Antonio Bustos-Merlo, Antonio Rosales-Castillo, Fernando Cobo, Carmen Hidalgo-Tenorio

**Affiliations:** 1 Department of Internal Medicine, Virgen de las Nieves University Hospital, 18014 Granada, Spain; antoniobustosmerlo@gmail.com (A.B.-M.); anrocas90@hotmail.com (A.R.-C.); 2 Department of Microbiology, Virgen de las Nieves University Hospital, 18012 Granada, Spain; fernando.cobo.sspa@juntadeandalucia.es; 3 Unit of Infectious Diseases, Virgen de las Nieves University Hospital, 18014 Granada, Spain

**Keywords:** *Mycoplasma hominis*, endocarditis, pacemaker lead

## Abstract

*Mycoplasma hominis* is a habitual colonizing microorganism of the lower genital tract but can exceptionally be the causal agent of blood culture-negative infective endocarditis (IE). Only 11 cases of this entity have been published to date. The study objectives were to describe the first case diagnosed in our center of IE by *M. hominis* on pacemaker lead and to carry out a narrative review. Among published cases of IE by this microorganism, 72.7% were male, with a mean age of 45 years and a history of valve surgery; the diagnosis was by culture (54.5%) or molecular technique (45.5%), and the prognosis was favorable in 72.7% of cases. The most frequently prescribed antibiotics were doxycycline, quinolones, and clindamycin.

## 1. Introduction

The *Mycoplasma* genus belongs to the *Mycoplasmataceae* family in the *Mollicutes* class. It includes a group of slow-growing bacteria species with no cell wall, characterized by a small genome and limited biosynthetic capacity. They are beta-lactam-resistant due to the absence of a cell wall, and their high nutritional demand makes them difficult to culture [1,2], although this problem has recently been solved by applying molecular techniques. They are often found as colonizers of mucosae, mainly respiratory and genitourinary [3,4]. *Mycoplasma hominis* was the first of various human pathogen species to be isolated. It colonizes the human genital tract and is transmitted by sexual contact [1]. It is not a pathogen in the adult vagina, being present in the vaginal/cervical secretions of around 50% of adult women [5]. However, it has increasingly been attributed to pathogenic action mechanisms at different levels and can be the cause of joint (periprosthetic, septic arthritis) [6], nervous system (drainage-related ventriculitis, neonatal meningitis) [7], respiratory [8], mediastinal [9], genitourinary (pyelonephritis, urethritis, bacterial vaginosis) [10], and even endovascular [11] infection. Reported pathogenicity mechanisms include enhanced adherence to eukaryotic cells (P50, P100), cell dissemination via certain proteins (e.g., Vaa), and local toxicity. Polymerase chain reaction (PCR) is frequently utilized for the microbiological identification of *M. hominis*, which is commonly susceptible to doxycycline and moxifloxacin [12].

We present the first reported case of *M. hominis* infective endocarditis (IE) on pacemaker lead and carry out a narrative review of previous reports on IE by this microorganism.

## 2. Materials and Methods

**Figure 1:**
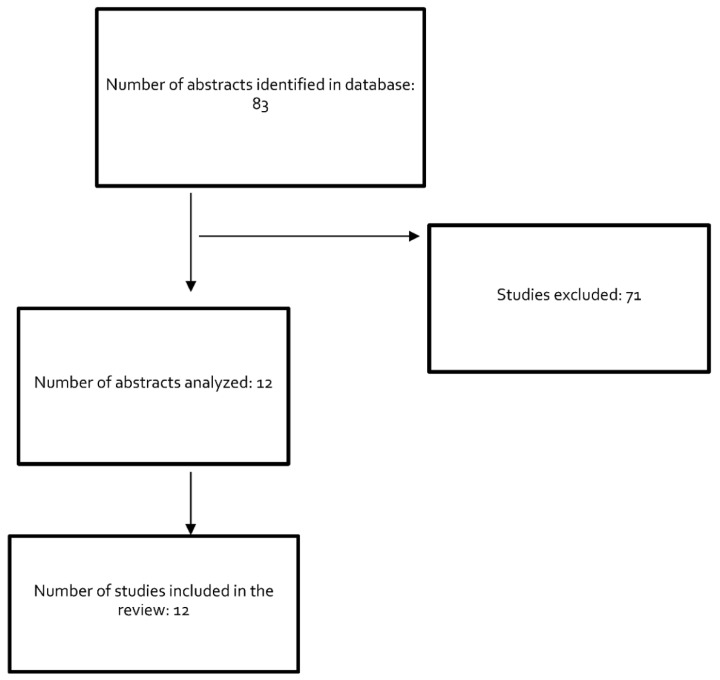
Flowchart of study selection for the narrative review.

A search was performed of the PubMed MEDLINE database up to 31 December 2021, including studies written in English or Spanish. Studies of other *mycoplasma* species and reports on cases without a confirmed diagnosis were excluded. Search terms were: “*mycoplasma* and endocarditis” (63 results), “*mycoplasma* and pacemaker” (5 results), “*mycoplasma* and prosthetic valve infection” (10 results), and “*mycoplasma* and endovascular infection” (5 results). The final review included 12 eligible studies describing 11 cases. Figure 1 depicts the study selection process.

## 3. Case Report

We report a case of blood culture-negative infective endocarditis (BCNIE) by *Mycoplasma hominis* in a 67-year-old male farmer with no family history of interest (early cardiovascular disease or sudden death) but a personal history of essential arterial hypertension, hypercholesterolemia, and asymptomatic hyperuricemia. He was under treatment with enalapril (20 mg/day), hydrochlorothiazide (50 mg)/amiloride (5 mg)/day, atorvastatin (80 mg/day), and apixaban (5 mg/12 h). He reported being an ex-smoker (CSI: 60 packs/year) and alcohol consumer (3 SDUs/day) with no known drug allergies.

On 28 April 2021, he was admitted to the cardiology department for sustained monomorphic ventricular tachycardia secondary to structural heart disease (inferolateral scar tissue in the left ventricle with no angiographic findings of significant coronary artery lesions). An implantable cardioverter-defibrillator (ICD) was placed after endocardial ablation, and he was discharged after three days.

On 7 May, 10 days later, he visited the emergency department for oppressive central chest pain radiating to the jaw, accompanied by vegetative symptoms. Physical examination evidenced blood pressure of 74/52 mmHg; heart rate of 104 bpm; respiratory rate of 22 rpm; peripheral oxygen saturation (SpO_2_) of 92% with inspired oxygen fraction (FiO_2_) of 21%; absence of fever; poor general state with mucocutaneous pallor; auscultated rhythmic heart sounds without murmurs and preserved vesicular murmur with no added sounds; soft, depressible abdomen non-painful to deep palpation; no megalies or signs of peritonism; preserved hydro-aerial sounds; no lower limb edemas; symmetrical pedal pulses; and no signs of deep venous thrombosis. Electrocardiogram (ECG) results were: sinus rhythm at 90 bpm; axis +30°; narrow QRS; Q wave in D III and aVF; and flattened T wave I, aVL, II, III, aVF, V4 to V6 (with no changes in results obtained at hospital discharge one week earlier). Venous blood gas values were pH 7.43, 23 mmHg pCO2, 15 mmol/L HCO3, and 2.5 mmol/L lactic acid. Blood analysis results are summarized in Table 1. There were no urine sedimentation abnormalities. Anteroposterior chest X-ray showed bilateral pleural effusion, more marked in the left hemithorax. Chest CT scan with intravenous contrast ruled out pulmonary thromboembolism, revealing mild bilateral pleural effusion (more marked on the left), moderate pericardial effusion of normal appearance, no evident thickening or enhancement of pericardial layers suggesting complications, and a slight increase in the density of subcutaneous cell tissue fat at the lower pacemakerpole. The patient was then transferred to the intensive care unit for suspicion of sepsis secondary to infection of the ICD generator pocket. Blood was drawn for culture in the absence of fever, and he was started on empirical treatment with meropenem (2 g/iv/8 h), daptomycin (10 mg/kg/iv/24 h), and clindamycin (600 mg/iv/8 h) alongside noradrenaline (target MAP of 65 mmHg) and rehydration therapy. Diagnostic thoracocentesis yielded 400 cc of serous fluid compatible with exudate according to Light’s criteria [pH 7.481, 9000 red blood cells, 1328 leukocytes with 80% polymorphonuclears, glucose 147 mg/dL, total proteins 3.7 g/dL (plasma 6.6 g/dL), and LDH 472 mg/dL (plasma 373 mg/dL)]. He then underwent cardiovascular surgery for ICD explantation, pericardiocentesis (aspirating 900 cc of exudate-like serohematic fluid), and debridement of the generator pocket. The pericardial fluid contained 580,000 red blood cells, 7400 leukocytes with 60% polymorphonuclears, glucose 0.1 mg/dL (plasma 116 mg/dL), and total proteins 5.6 g/dL (plasma 6.2 g/dL). The fluid was studied in the microbiology laboratory using SeptiFast (LightCycler^®^ SeptiFast Test Mgrade; Roche Diagnostics GmbH, Mannheim, Germany), a PCR test for bacteria and fungi identification, and was found to be positive for *Mycoplasma* sp. Accordingly, the antibiotic regimen was changed to moxifloxacin (400 mg/24 h) and doxycycline (100 mg/12 h) intravenously for the first week and then orally.

Four sets of hemocultures were obtained at different times, and SeptiFast identified *M. hominis* in one of these. Pleural fluid culture was negative. Cultures of pericardial fluid, “pocket” wound exudate, and ICD lead samples all evidenced the growth of *M. hominis,* which was resistant to erythromycin and clindamycin and susceptible to levofloxacin, ciprofloxacin, tetracycline, and doxycycline. Negative results were obtained for methicillin-resistant S. aureus colonization from the nasal exudate samples and for multi-drug resistant bacteria from the culture of perianal exudate samples. Transthoracic echocardiography study at five days post-pericardiocentesis evidenced no anatomical or functional valvular abnormalities but the persistence of the mild pericardial effusion, while the right cavities showed no sign of hemodynamic compromise.

A new ICD was implanted after the patient achieved hemodynamic stabilization and normalization of acute phase reactants 12 days after the start of antibiotic therapy (Table 1). There were no complications, and the patient had a favorable outcome, being discharged at two weeks post-implantation with no symptoms (NYHA functional class I) and complete resolution of the pericardial effusion. The combined antibiotic treatment was continued for a further two days post-discharge. At 10 months post-discharge, the patient remained asymptomatic with no new ICD-related incidents.

## 4. Discussion

The narrative review yielded 11 cases of IE by *M. hominis.* The data summarized in Table 2 show that 72.7% of the patients were male with a mean age of 45 years (range, 4–74 years) and out of the 11 patients, 10 had *a* history of heart surgery for valve replacement, valve repair, or congenital malformation [13,14,15,16,17,18,19,20,21,22,23], as in the present case.

The underlying pathogenic mechanism of endocarditis by *M. hominis* appears to be nosocomial, possibly secondary to bacteremia caused by the instrumentalization of colonized areas before, during, or after surgery. The median time interval between heart surgery and symptom onset was six months (range, 1–12 months).

The most frequent clinical signs/symptoms in the published cases were fever and valve insufficiency [13,14,15,16,17,18,19,20,21,22,23]. Serial blood cultures were negative in all 11 patients. *M. hominis* was identified by culture in six cases (in combination with molecular analysis in three of these) and by molecular analysis (16 s ribosomal DNA PCR) of the explanted valve in the remaining five [13,14,15,16,17,18,19,20,21,22,23].

Blood culture plays an essential role in the diagnosis and treatment of IE, and a major diagnostic challenge is therefore posed by blood culture-negative infective endocarditis (BCNIE) [24], reported to represent between 2.5 and 70% of all endocarditis cases in different countries and series [25]. It is essential to consider other causes in patients with IE and negative blood cultures after ruling out an infectious etiology [26]. Besides serology for the detection of fastidious agents such as *Coxiella burnetii* and *Bartonella* spp. [26], valve biopsy is the most useful diagnostic approach, especially with the application of histologic and broad-range PCR techniques, when available [27].

*Mycoplasma hominis* does not Gram stain, it has limited biosynthetic capacity, and it is difficult to isolate in culture medium1. In addition, the characteristic “fried egg” (0.3–0.6 nm) appearance of colonies in culture hampers their ready visualization [25]. Currently, PCR is successfully used to study the extracted valve, offering elevated specificity and sensitivity values [27]. In the present case, the study of blood cultures with real-time multiplex PCR (Septifast) yielded a diagnosis only a few hours after establishing clinical suspicion, allowing the early administration of targeted antibiotic therapy.

After blood samples had been drawn for culture, all patients received empirical antibiotic treatment. Seven (63.6%) were prescribed combined antibiotic treatment with three drugs, most frequently associating a glycopeptide or lipopeptide with beta-lactam and aminoglycoside [13,16,17,18,21,22,23]. The other four received a combination of glycopeptide with aminoglycoside, beta-lactam with aminoglycoside, glycopeptide with beta-lactam, or glycopeptide with lipopeptide [14,16,19,20]. The present patient was treated with a combination of beta-lactam, lipopeptide, and macrolide.

After the detection of *M. hominis*, nine of the eleven patients (81.8%) [13,14,15,17,18,19,20,21] were prescribed targeted antibiotic treatment, one died during the immediate postoperative period, and the remaining patient was lost to the follow-up [16,22]. Only one of the empirical treatments initially received by the patients was active against *M. hominis* [16]. The antibiotics most frequently administered in targeted therapy were doxycycline (7/9 cases, 77.8%) [13,15,17,18,19,20,21], quinolones (6/9 cases, 66.7%) [16,17,19,20,21,23], and clindamycin (4/9 cases, 44.4%) [13,16,18,20]. All patients were treated with a combination of antibiotics except for one prescribed monotherapy with doxycycline15.

The duration of treatment ranged between 4 and 10 weeks (median of 8 weeks, mean of 9.5 weeks). Eight patients (72.7%) had a favorable outcome, including two who required heart transplantation [13,23], two patients (18.2%) died [14,16], and one was lost to the follow-up [22].

The intrinsic structural characteristics of *M. hominis* render it resistant to the antibiotics usually administered against IE, including macrolides, aminoglycosides, beta-lactams, and cotrimoxazole. The treatment of choice is doxycycline (100 mg/12 h), quinolones (moxifloxacin [400 mg/day], levofloxacin [500 mg/day]), or clindamycin (300 mg/8 h) [12]. The optimal antibiotic regimen for endocarditis by *M. hominis* has not been established, although most authors recommend a single antibiotic therapy cycle of 6–8 weeks alongside valve replacement [13,14,15,16,17,18,19,20,21,22,23]. One patient who received doxycycline for 8 weeks required heart transplantation for acute heart failure due to recurrent perivalvular insufficiency [13].

In conclusion, *M. hominis* is rarely the cause of IE but should be contemplated in cases of BCNIE, especially in individuals with a recent history of cardiac surgery or device implantation. Although the clinical manifestations are non-specific, this entity should be considered in such patients in the presence of fever, clinical bacteremia, and/or signs/symptoms of heart failure not previously experienced. Clinical suspicion is also strengthened by the presence of elevated acute phase reactants (C-reactive protein, procalcitonin, leukocytosis) early after cardiac surgery with no evident focus of infection, especially when blood cultures are negative [19].

We highlight the diagnostic usefulness of molecular techniques, initially applied in blood samples, allowing the identification and recovery of a proportion of cases with negative blood cultures. Nevertheless, the definitive diagnosis is usually obtained through the identification of *M. hominis* in samples of valves or endovascular prosthetic material [18].

A more rapid diagnosis permits the earlier administration of targeted treatment, with doxycycline and quinolones as the drugs of choice. This has important prognostic implications, given the intrinsic resistance of *M. hominis* to the antibiotics habitually prescribed against IE produced by staphylococci and streptococci [14].

## Figures and Tables

**Table 1:** Patient analytics at infection onset and after 12 days of antibiotic treatment.

Variable	Reference Range in Our Hospital	At Hospital Admission	At Intensive Care Unit Admission	12 Days after Initiation of Antibiotherapy
Hemoglobin (g/dL)	12.5–17.2	11.2	10.5	10.6
Hematocrit (%)	37–49	35.6	29.2	32.8
Leukocyte count (μL)	3600–10,500	13,060	19,000	5840
Absolute neutrophil count (μL)	1500–7700	11,580	17,000	3030
Absolute lymphocyte count (μL)	1–1400	610	1330	1770
Absolute monocyte count (μL)	100–900	680	510	680
Platelet count (μL)	130,000–370,000	326,000	265,000	364,000
Prothrombin time (%)	75–130	73.1	49	69
Fibrinogen (mg/dL)	170–450	900	773.5	335
D-Dimer (mg/L)	0–0.5	3.19		
Glucose (mg/dL)	75–115	183	149	87
Urea (mg/dL)	18–37.7	92	129	42
Creatinine (mg/dL)	0.67–1.17	2.03	3.74	1.22
Total bilirubin (mg/dL)	0.3–1.2	1.23	1.67	0.8
Aspartate transaminase (U/L)	10–50	67	5425	22
Alanine transaminase (U/L)	1–50	95	4950	62
Gamma-glutamyl transferase (U/L)	1–55	462	336	33
Alkaline phosphatase (U/L)	30–120	219	197	84
Lactate dehydrogenase (U/L)	10–248	362	5515	253
Sodium (mEq/L)	136–145	137	141	143
Potassium (mEq/L)	3.50–5.10	4.90	4.60	4.30
Ferritin (ng/mL)	20–250	504.5		31
NT-proBNP (pg/mL)	10–300	1430	5450	328
C-reactive protein (mg/L)	0.1–5	142.9	320	2.2
Procalcitonin (ng/mL)	0.02–0.5	9.57	>100	
High-sensitivity troponin I (pg/mL)	2.3–19.8	18.8	88.8	

**Table 2:** Cases of endocarditis and *M. hominis* documented in the literature.

Cases	Reference	Age (Years)- Sex	History of Cardiac Surgery	Time Elapsed from Surgery to Diagnosis	Species Isolated (Method)	Positive Sample	Valve Involvement	Empirical Treatment	Targeted Treatment	Outcome
1	Cohen et al. [11] DiSesa et al. [13]	25-F	Aortic and mitral valve replacement	3 months	*Mycoplasma hominis* (culture)	Mitral annulus sample	Mitral and aortic	Vancomycin, gentamycin, and ampicillin	Clindamycin and rifampicin iv. (6 w) Oral doxycycline (4 w)	Rescue with orthotopic heart transplantation. Survives
2	Blasco et al. [14]	46-M	Mitral valve replacement	15 days	*Mycoplasma hominis* (culture)	Prosthetic valve sample	Dehiscence of the mitral prosthesis	Vancomycin and amikacin	Not reported	Death
3	Fenollar et al. [15]	33-M	Mitral valve annuloplasty	6 months	*Mycoplasma hominis* (16S rDNA PCR)	Cardiac valve specimen	Dehiscence of mitral plasty	Amoxicillin and gentamycin	Doxycycline (4 w)	Survives
4	Domínguez et al. [16]	4-F	Biventricular repair	22 days	*Mycoplasma hominis* (culture and 16S rDNA PCR)	Pleural fluid, blood sample, and myocardium sample	Right atrial vegetation and perivalvular abscess	Vancomycin, meropenem, levofloxacin, gentamycin, and fluconazole	Clindamycin and levofloxacin	Death
5	Hidalgo-Tenorio et al. [17]	48-M	Aortic valve replacement	2 months	*Mycoplasma hominis* (culture)	Valve sample	Dehiscence of aortic plasty and acute regurgitation	Vancomycin, gentamycin, and cefepime	Doxycycline and levofloxacin (8 w)	Survives
6	Jamil et al. [18]	40-M	Aortic and mitral valve replacement	9 years	*Mycoplasma hominis* (16S rDNA PCR and culture)	Valve sample	Partial mitral prosthesis dehiscence with severe paraprosthetic regurgitation	Vancomycin, ciprofloxacin, and rifampicin	Doxycycline and clindamycin (8 w)	Survives
7	Hussain et al. [19]	57-M	Aortic valve replacement and mitral valve decalcification	1 year	*Mycoplasma hominis* (16S rDNA PCR)	Valve sample	Aortic regurgitation	Vancomycin and ceftriaxone	Doxycycline iv and oral moxifloxacin (duration ND)	Survives
8	Gagneux-Brunon et al. [20]	74-M	Aortic and mitral valve replacement	6 months	*Mycoplasma hominis* (16S rDNA PCR and culture)	Cardiac valve sample	Dehiscence of aortic prosthesis	Vancomycin, Linezolid, and Daptomycin (after suspending linezolid).	Moxifloxacin (2 days). Clindamycin and doxycycline (9 w)	Survives
9	Romeu Prieto et al. [21]	54-M	Aortic valve replacement	7 months	*Mycoplasma hominis* (16S rDNA PCR)	Valve sample	Massive aortic regurgitation	Daptomycin, ceftriaxone, and gentamicin.	Doxycycline and levofloxacin (8 w)	Survives
10	Kotaskova et al. [22]	ND-F	Tricuspid valve replacement	1 year	*Mycoplasma hominis* (16S rDNA PCR) and *Sneatha sanguinegens*	Valve sample	Massive tricuspid regurgitation	Amoxicillin/clavulanic acid, ampicillin and gentamicin	Not reported	Loss to follow up. Death
11	Givone et al. [23]	28-M	Aortic valve and root replacement	13 months	*Mycoplasma hominis* (16S rDNA PCR)	Aortic tissue specimen	Para-aortic pseudoaneurysm and severe aortic insufficiency	Daptomycin, ampicillin, and anidulafungin	Moxifloxacin (8 w)	Survives. Heart transplant

## Data Availability

The data presented in this study are available in the main text.
